# Adding robustness to rigor and reproducibility for the three Rs of improving translational medical research

**DOI:** 10.1172/JCI173750

**Published:** 2023-09-15

**Authors:** Michael P. McGill, David W. Threadgill

**Affiliations:** 1 Interdisciplinary Graduate Program in Genetics and Genomics,; 2 Department of Cell Biology and Genetics,; 3 Department of Nutrition, and; 4 Department of Biochemistry and Biophysics, Texas A&M University, College Station, Texas, USA.

## Introduction

To improve advances in scientific research, the National Institutes of Health has emphasized rigor and reproducibility, where rigor ensures “robust and unbiased experimental design, methodology, analysis, interpretation, and reporting of results,” while reproducibility is evident when data can be “reproduced by multiple scientists” (1). However, even in rigorous and reproducible research, there is increasing evidence that results using genetically homogeneous preclinical models for disease can fail to translate to a genetically diverse human patient population. The relative ease with which results can be gathered using a single model often leads researchers to discount the possibility that the results may not be representative of more diverse genetic backgrounds, reducing the translational potential for humans. To improve translation, we propose as one solution that a robustness test should be considered to confirm that results are “robust across heterogeneous genetic contexts,” thereby improving prediction of likely responses in heterogeneous patient populations. Furthermore, robustness approaches could be leveraged to identify biomarkers that prognosticate likely responders, heightening public health outcomes and alleviating financial burden. This general concept pertains to all genetically homogeneous preclinical models as well as large, genetically ill-defined outbred animals used in small numbers for safety testing, but mice will be used as the exemplar given their extensive use in modeling therapeutic efficacy in human diseases.

## Origin of translational failures

**Figure 1:**
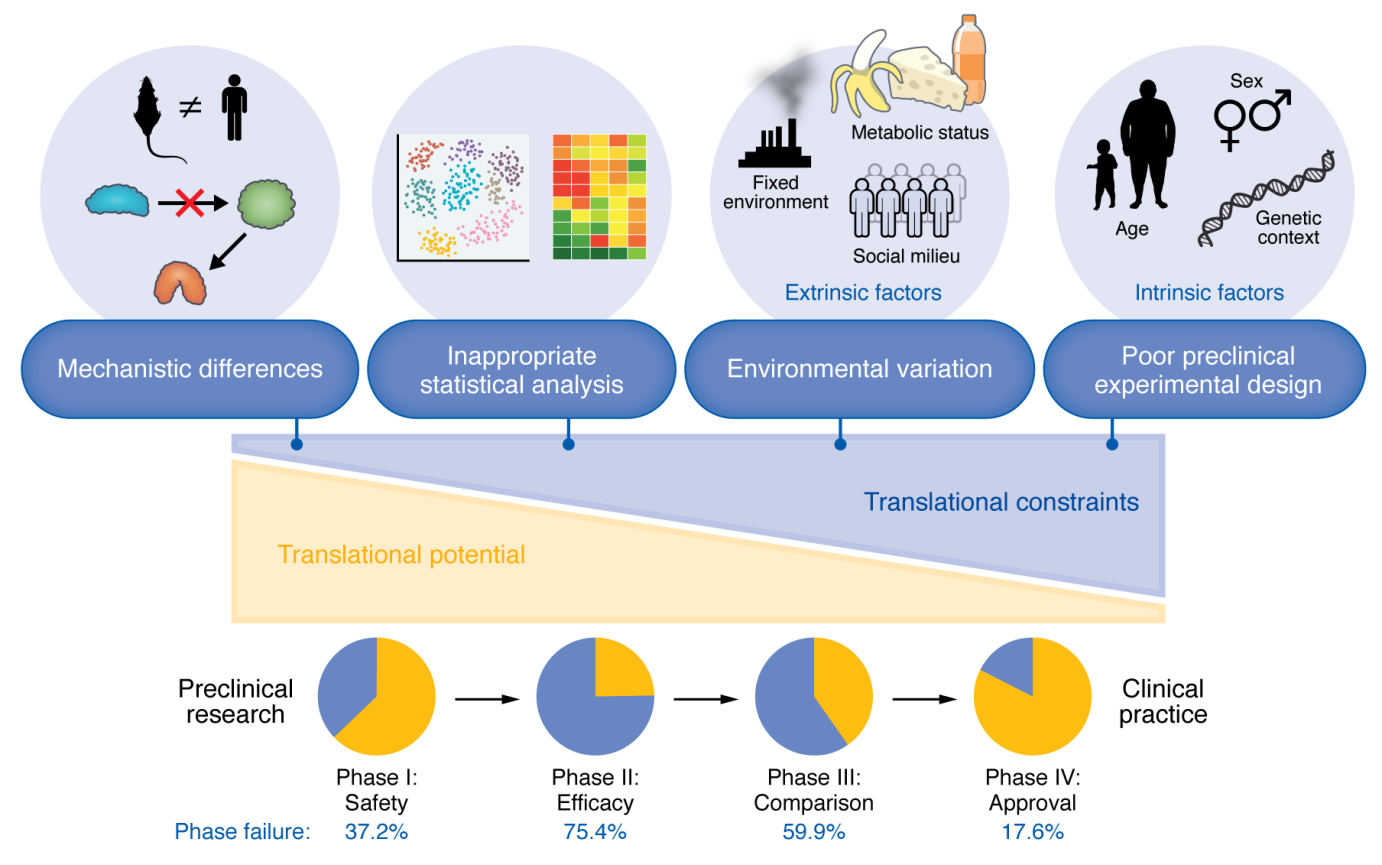
Four constraints contribute to translational failures of preclinical studies. Rigorous, reproducible, and robust experimental designs can ameliorate experimental constraints that have led to high rates of translational failure. Preclinical results can be affected by extrinsic and intrinsic factors that can be better modeled to improve translational success by embracing heterogeneity as an inherent biological phenomenon.

Therapeutic candidates tested in clinical trials primarily originate from results gathered using preclinical models. However, preclinical results frequently fail to be reproduced in the clinical setting. Only 11% of published “landmark” findings in oncology were validated in clinical trials (2), consistent with other disease areas, due to an inability to demonstrate safety and efficacy (Figure 1). Aside from the impact on enrolled patients, these failures produce an estimated economic burden of roughly $45 million per failed clinical trial.

Variability in response to experimental therapeutics among patients is a common concern during clinical trials, yet preclinical models are almost always genetically and environmentally homogeneous. An analysis of over 2,000 drugs found that common genetically homogeneous preclinical species are inconsistent predictors of toxic responses in humans (3). However, retrospective studies using heterogeneous models support their utility for predicting therapeutic safety. For example, heterogeneous preclinical populations have predicted adverse responses to phosphatidylinositol 3-kinase inhibitors for the treatment of chronic lymphocytic leukemia and follicular lymphoma (4) and liver injury during the use of vasopressin V_2_-receptor antagonists in the treatment of hyponatremia (5). These discoveries could have informed clinical trial design if they had been identified proactively during the preclinical phase. In general, four areas contribute to translational failures from homogeneous preclinical models to heterogeneous human patient populations (Figure 1).

### Mechanistic differences.

Mechanistic differences between mice and humans can limit translation but are still informative for understanding molecular mechanisms. Classical examples include the loss of hypoxanthine-guanine phosphoribosyl transferase (HPRT) to model Lesch-Nyhan syndrome (6) and disruption of the tumor-suppressor RB transcriptional corepressor 1 (RB1) to model retinoblastoma (7). Deficiency of the X-linked purine salvage pathway gene *HPRT* causes Lesch-Nyhan syndrome in humans, which is characterized by self-injurious behavior and behavioral abnormalities. However, the development of *Hprt*-deficient mice resulted in no phenotypic neurobehavioral abnormalities (8). Further investigation showed that mice can use an alternative purine salvage pathway utilizing adenine phosphoribosyl transferase (APRT). Although subsequent treatment with an APRT inhibitor induced persistent self-injurious behavior in *Hprt*-deficient mice (9), this was not replicable in *Hprt^–/–^* *Aprt^–/–^* double-mutant mice (10). The failure of the *Hprt*, *Aprt*–deficient mice to generate any phenotypic abnormalities resembling the human disease suggests still-unknown species and/or genetic background-dependent differences between mice and humans.

Retinoblastoma in humans is the result of deficiency of RB1, and individuals who inherit one defective copy of *RB1* frequently develop retinoblastoma early in life. Unlike children bearing germline *RB1* mutations, heterozygous *Rb1* mutant mice fail to develop retinoblastoma. It was later discovered that the related protein RBL1 (also known as p107) protects against tumorigenesis in mice and mutation in both *Rb1* and *Rbl1* is needed for retinoblastoma development in mice (11). The use of mice in translational studies evaluating mechanisms that differ between mice and humans can, even if used in a rigorous and reproducible study, lead to translational failures. Notably, neither Lesch-Nyhan syndrome nor retinoblastoma mouse models have been evaluated on more than one genetic background. However, extensive analysis of these diseases in humans now shows a continuum of disease severity ranging from clinically insignificant to very severe for Lesch-Nyhan (12) and low to high penetrance for retinoblastoma (13), likely related to genetic differences among patients that have not been explored in mice.

### Inappropriate statistical analysis.

Inappropriate statistical analysis can also lead to translational failures. Phenotypic traits observed in preclinical models may present similarities or differences with humans that do not exist due to inappropriate comparisons. Previous comparisons of inflammatory responses between humans and C57BL/6J (B6J) mice revealed significant differentially expressed genes (14). However, a low correlation in gene expression changes was observed between humans and mice, leading to the conclusion that mice do not accurately reflect human inflammatory diseases. Using the same gene expression data sets, another group arrived at an opposing conclusion (15). Instead of analyzing sets of genes that were changed in human inflammatory conditions regardless of expression changes in mice, researchers analyzed genes whose expression levels were significantly changed in both humans and mice. This led to the conclusion that gene expression patterns in mice do recapitulate those seen in human conditions and that mice faithfully model human inflammatory disease. Even without considering the questionable comparison of heterogeneous humans with a homogeneous mouse model, these contrasting analyses demonstrate the potential impact of different statistical approaches on translational interpretation.

### Environmental variation.

Environmental variation is a major confound in patient populations. Despite tight environmental control in preclinical studies, mice can display variable behavioral and molecular responses even when genetically identical (16). Phenotypic plasticity is complex and fluctuates depending on extrinsic factors, such as the fixed environment and social milieu, in addition to intrinsic factors, including genetic background and sex. Consequently, environmental variation can lead to poor reproducibility across laboratories and facilities and, in some cases, translational failures. While these environmental differences are difficult to control and can confound results in reproducibility studies, environmental variability could be leveraged to ensure robust results. Dividing large animal experiments into several smaller experiments spread over time has been shown to increase reproducibility compared with conventional experimental designs (17). Multilaboratory, environmentally heterogeneous experimental designs can also increase robustness by capturing biologically relevant variation that is largely absent under highly standardized conditions (18). A systematic analysis of the influence of gene-by-environment interactions on phenotypes spanning asthma, type 2 diabetes mellitus, obesity, anxiety, and immunological, biochemical, and hematological phenotypes using genetically heterogeneous mice demonstrated that genetic variation explains more observed phenotypic variation than the environment in over 75% of phenotypes analyzed, with genetics accounting for roughly 90% of the observed variation in some physiological phenotypes (19). Furthermore, subsequent analysis of environmental and physiological covariates indicated that sex is also influential and was involved in each case when a covariate explained over 25% of the observed variation. In cases where preclinical studies are highly standardized and environmental conditions are tightly controlled, genetically homogeneous models serve as poor models for the genetically and environmentally heterogeneous human patient population.

### Poor preclinical experimental designs.

Poor preclinical experimental designs that are not properly configured to translate results from homogeneous model systems to heterogeneous patient populations likely result in many translational failures. Although the experimental design may be sound for the scientific question at hand, the design can still be poor if the ultimate goal of the study is translation to heterogeneous patient populations. To generate robust conclusions, genetic heterogeneity of the human patient population should be captured in preclinical models. By modeling heterogeneity of the disease state in preclinical studies, more robust conclusions can be reached, illuminating the spectrum of patients likely to benefit from specific clinical interventions.

## Impact of genetic heterogeneity on interpreting results from preclinical models

Studies using the B6J and C57BL/6NJ (B6N) substrains highlight the impact of genetic background on experimental interpretation. Using similar knockout alleles in mitogen-activated protein kinase 9 (*Mapk9*), one group reported that MAPK9 prevents acetaminophen-induced hepatotoxicity (20), while another group reported MAPK9 contributes to acetaminophen-induced hepatotoxicity (21). A subsequent study showed the contradictory results were due to differences in genetic context, with MAPK9 being hepatoprotective in B6J mice and hepatotoxic in B6N mice (22). Even in genetically similar strains such as B6J and B6N, phenotypic heterogeneity can inform translation to the clinical setting.

Our group discovered one of the first examples of the importance of genetic context using genetic ablation of the epidermal growth factor receptor (*Egfr*). EGFR is commonly expressed in advanced colorectal cancers (CRCs), leading to one of the first molecular targeted therapies. However, when EGFR inhibitors were tested in clinical trials, they demonstrated modest to no efficacy. Genetic background profoundly affects the robustness of EGFR inhibition in preclinical CRC models, as most colon tumors are EGFR dependent in B6J mice while being EGFR independent in humans (23), providing evidence for the observed lack of translation.

Similarly, ablation of *Erbb3* in the intestinal epithelium was initially reported to protect against CRC, which contributed to the rationale for developing pharmacological inhibitors against ERBB3. However, clinical trials with inhibitors blocking ERBB3 had little to no efficacy in the colon and showed a tendency toward promoting tumor progression (24). A subsequent preclinical robustness assay using different genetic backgrounds showed that deletion of *Erbb3* results in a significant reduction in polyp number when performed on a 129S1/SvImJ background, but increased polyp number in B6J mice (25). These studies suggested that the outcomes of ERBB3 clinical trials would have been predicted using a robustness assay, which could have led to a more informed clinical trial.

## Ensuring more robust conclusions from preclinical models

A large portion of translational failures could likely be mitigated by using better experimental designs to evaluate the robustness of results before advancing to clinical trials. As a general recommendation, testing results for robustness in at least one additional genetic background from the same or different species that models the human disease, now possible with recent advancements in CRISPR-Cas9, will increase the amount of genetic variability captured in preclinical studies and illuminate the potential limitations of clinical trials. While this may seem burdensome and will add experimental cost, these approaches will provide the ability to predict heterogeneous patient responses, the potential for discovering novel genetic modifiers of therapeutic targets, and the foundation to reveal biomarkers of response needed to partition patient populations during clinical trials. Although determining when a robustness test should be performed in the preclinical setting will usually not be obvious from the examples above, the use of such approaches before particularly expensive clinical trials might warrant the additional cost to mitigate financial risk and ultimately enhance the likelihood of successful clinical trials. The example of ERBB3 inhibitors above shows where robust preclinical designs would have anticipated mixed clinical responses.

The oversimplified approach using genetically homogeneous models or small numbers of genetically ill-defined large animals has led to a perception that many studies do not translate to the clinic even though two-species tests are being evaluated to be replaced by one-species tests for safety testing (26). Admittedly, other approaches such as panels of patient samples, could conceivably improve the translational merit of preclinical studies. Nonetheless, by expanding the National Institutes of Health’s directive on rigor and reproducibility to the 3Rs of rigor, reproducibility, and robustness that embraces genetic heterogeneity as an inherent biological phenomenon, translational studies will have a greater impact by improving clinical study design, ultimately leading to improved health and reduction of disease.

## Version 1 09/15/2023

Electronic publication

## References

[1] Hofseth LJ (2018). Getting rigorous with scientific rigor. Carcinogenesis.

[2] Begley CG, Ellis LM (2012). Drug development: raise standards for preclinical cancer research. Nature.

[3] Bailey J (2014). An analysis of the use of animal models in predicting human toxicology and drug safety. Altern Lab Anim.

[4] Mosedale M (2019). Identification of candidate risk factor genes for human idelalisib toxicity using a collaborative cross approach. Toxicol Sci.

[5] Mosedale M (2017). Editor’s highlight: candidate risk factors and mechanisms for tolvaptan-induced liver injury are identified using a collaborative cross approach. Toxicol Sci.

[6] Finger S (1988). Behavioral and neurochemical evaluation of a transgenic mouse model of Lesch-Nyhan syndrome. J Neurol Sci.

[7] Jacks T (1992). Effects of an Rb mutation in the mouse. Nature.

[8] Jinnah HA (1994). Tetrahydrobiopterin deficiency and dopamine loss in a genetic mouse model of Lesch-Nyhan disease. J Inherit Metab Dis.

[9] Wu CL, Melton DW (1993). Production of a model for Lesch-Nyhan syndrome in hypoxanthine phosphoribosyltransferase-deficient mice. Nat Genet.

[10] Engle SJ (1996). HPRT-APRT-deficient mice are not a model for lesch-nyhan syndrome. Hum Mol Genet.

[11] Robanus-Maandag E (1998). p107 is a suppressor of retinoblastoma development in pRb-deficient mice. Genes Dev.

[12] Torres RJ (2012). Update on the phenotypic spectrum of Lesch-Nyhan disease and its attenuated variants. Curr Rheumatol Rep.

[13] Alekseeva EA (2021). Parental origin of the *RB1* gene mutations in families with low penetrance hereditary retinoblastoma. Cancers (Basel).

[14] Seok J (2013). Genomic responses in mouse models poorly mimic human inflammatory diseases. Proc Natl Acad Sci U S A.

[15] Takao K, Miyakawa T (2015). Genomic responses in mouse models greatly mimic human inflammatory diseases. Proc Natl Acad Sci U S A.

[16] Freund J (2013). Emergence of individuality in genetically identical mice. Science.

[17] von Kortzfleisch VT (2020). Improving reproducibility in animal research by splitting the study population into several ‘mini-experiments’. Sci Rep.

[18] Voelkl B (2018). Reproducibility of preclinical animal research improves with heterogeneity of study samples. PLoS Biol.

[19] Valdar W (2006). Genetic and environmental effects on complex traits in mice. Genetics.

[20] Bourdi M (2008). Protective role of c-Jun N-terminal kinase 2 in acetaminophen-induced liver injury. Biochem Biophys Res Commun.

[21] Nakagawa H (2008). Deletion of apoptosis signal-regulating kinase 1 attenuates acetaminophen-induced liver injury by inhibiting c-Jun N-terminal kinase activation. Gastroenterology.

[22] Bourdi M (2011). Mispairing C57BL/6 substrains of genetically engineered mice and wild-type controls can lead to confounding results as it did in studies of JNK2 in acetaminophen and concanavalin A liver injury. Chem Res Toxicol.

[23] Mantilla-Rojas C (2021). A molecular subtype of colorectal cancers initiates independently of epidermal growth factor receptor and has an accelerated growth rate mediated by IL10-dependent anergy. Oncogene.

[24] Hill AG (2018). Phase II study of the dual EGFR/HER3 Inhibitor Duligotuzumab (MEHD7945A) versus cetuximab in combination with FOLFIRI in second-line *RAS* wild-type metastatic colorectal cancer. Clin Cancer Res.

[25] Mantilla Rojas C (2021). Epithelial-specific ERBB3 deletion results in a genetic background-dependent increase in intestinal and colon polyps that is mediated by EGFR. PLoS Genet.

[26] Prior H (2020). Opportunities for use of one species for longer-term toxicology testing during drug development: A cross-industry evaluation. Regul Toxicol Pharmacol.

